# Knowledge and Attitudes of Saudi Medical Students Toward Forensic Medicine as a Subspecialty: A Cross-Sectional Study

**DOI:** 10.7759/cureus.73096

**Published:** 2024-11-05

**Authors:** Wafi B Alotaibi, Raghad H Abduljabbar, Raghad M Al-Awn, Maha A Albakr, Safia M Binshihon, Alhassan H Hobani, Mohammad S Alnejaidi, Mohammad E Mahfouz

**Affiliations:** 1 College of Medicine, Taif University, Taif, SAU; 2 Medicine and Surgery, King Khalid University Hospital, Abha, SAU; 3 College of Medicine, King Saud Bin Abdulaziz University, Riyadh, SAU; 4 Forensic Medicine, Forensic Medicine Centre, Ministry of Health, Jeddah, SAU; 5 College of Medicine, Jazan University, Jazan, SAU; 6 College of Medicine, King Abdulaziz University, Jeddah, SAU; 7 Surgery, Taif University, Taif, SAU

**Keywords:** forensic, forensic medicine, justices, knowledge, medical students

## Abstract

Background: The field of forensic medicine, also known as medical jurisprudence, plays a vital role in civil and criminal legal proceedings by applying medical principles to establish facts. Despite its critical importance, there is a significant shortage of experts in this subspecialty. Understanding the factors influencing medical students' interest in forensic medicine is crucial.

Methods: The study was an observational cross-sectional conducted among medical students at Taif University, Saudi Arabia. Data was collected through online questionnaires distributed through social media applications (WhatsApp, Twitter, and Facebook) through Google Forms. Data was analyzed using Statistical Package for the Social Sciences (IBM SPSS Statistics for Windows, IBM Corp., Version 27.0, Armonk, NY) to obtain important insights.

Results: The majority of the medical students (65.2%) demonstrated good knowledge of forensic medicine. Furthermore, an overwhelming majority, 94.6%, agree that forensic medicine is a fundamental part of the medical field. Further, our study showed that 87% of the medical students have received formal education related to forensic medicine, though only 38% feel that the education was adequate. In comparison, nearly half (44.6%) of students felt that the education they received regarding forensic medicine was inadequate. Almost three-quarters of the medical students (70.7%) believe it would be beneficial to incorporate the forensic medicine module in their curriculum. The key motivating factors for choosing forensic medicine as a career were personal interest (39.1%) and the nature of the work (32.6%). Gender was not significantly associated with knowledge of forensic medicine (p-value<0.05).

Conclusion: Medical students strongly recognize the importance of forensic medicine and support its inclusion in the curriculum. However, many feel underprepared for medico-legal responsibilities, indicating a gap in current education. Personal interest and the nature of the work are key motivators, with lectures playing a crucial role in career decisions. The study found no significant gender differences in knowledge or attitudes toward forensic medicine. It recommends that medical schools incorporate a comprehensive forensic medicine module to address these educational gaps.

## Introduction

Forensic medicine, commonly known as medical jurisprudence, is that department of medicine that applies its principles to civil and criminal judicial proceedings and helps in the establishment of facts based on medicine [1]. This goes from medicolegal autopsies to examinations in cases - for example, of domestic abuse and rape - having an important contribution to the administration of justice [1,2]. Despite the critical nature of the field, the forensic medicine subspecialty has an acute shortage of experts, making it pertinent to understand the factors that influence medical students to take up the medical jurisprudence subspecialty [3].

Medical education has advanced rapidly in the Kingdom of Saudi Arabia, but little achievement is seen in this whole emphasis on integrating forensic medicine into the medical curriculum. Recent studies indicate that the overall level of knowledge and interest in medical students about the roles and responsibilities of forensic medicine is found to be poor [4]. There lies a grey area in this lack of knowledge and interest level, as it finds an important role in legal and healthcare systems [5].

The cross-sectional surveys in 2013 and 2016 among the students of the faculties of forensic medicine have highlighted a deep unawareness of the students regarding the everyday tasks of forensic physicians and the implications for the larger field [4-7]. More than three-quarters of students felt under-prepared for the future role they would take when they were involved in forensic medicine; hence, this really emphasizes a big gap between the current educational program and specialty requirements [8]. These findings are worrisome because they reflect potential difficulties in attracting future professionals to a field that is critical both for healthcare delivery and legal adjudication [9]. This will necessitate due attention to the attitudes and knowledge of medical students towards forensic medicine considering these challenges across wider demographics within the Kingdom of Saudi Arabia.

Our study will therefore try to fill this gap by assessing the level of knowledge and attitudes toward forensic medicine, in addition to the factors that are most influential, among students of Taif University. We conducted an inclusive survey to find out the level of interest and comprehension among medical students in the field of forensic medicine, hoping it might tell us what to do to improve the quality of educational exposure and raise activity in this important branch of medicine. It is in this light that the study narrows down, hence, not only adding to the academic literature but also offering some actionable insights to medical educators and policymakers. Incorporation of solid forensic medicine modules into the curriculum might attract a larger share of students towards specialization in the field, which would further strengthen the interface of healthcare systems with judicial processes in Saudi Arabia.

## Materials and methods

Study design

This research utilized a cross-sectional design and was carried out between August 2023 and November 2023. The study involved the medical students at Taif University, Taif, Saudi Arabia. The study aimed to evaluate the knowledge and attitudes of medical students toward forensic medicine at Taif University.

Inclusion and exclusion criteria

The study included all medical students at Taif University, irrespective of gender, who expressed willingness to participate. Non-medical students at Taif University and other non-student staff working in the medical department were excluded from the study.

Sampling technique

The study employed a convenient sampling technique, whereby students were enrolled based on their availability and willingness to participate at the time of data collection.

Sample size

To determine sample size, we used the Cochrane sample size approach, which is given below;

\[ n = \frac{Z^2 (1 - p)}{d^2} \]

where n is the sample size, Z is the critical statistic for a 95% confidence interval the anticipated knowledge is 60% and d is the margin of error, set at 10%. The minimum acceptable sample size was 92 [10].

Data collection tools and procedures

The researchers devised a questionnaire tailored to the study's aims and objectives, drawing from pertinent literature. To gauge its feasibility, understandability, and readability, a pilot test involving 10 respondents was conducted. The resulting Cronbach’s alpha, at 0.83, signified strong internal consistency. The students involved in the pilot study were distinct from those included in the main study. After the pilot study feedback, there were subsequent adjustments to the questionnaire. The final questionnaire was disseminated online via WhatsApp and Telegram groups to the medical students at Taif University through Google Forms. These groups were part of specialized student clubs and networks focused on academic and extracurricular activities within the medical school, ensuring the questionnaire reached the intended audience effectively. Comprising three sections, the questionnaire first delved into socio-demographic variables. The second section, comprising 11 questions, probed respondents' knowledge of forensic medicine and its associated responsibilities. Lastly, the third section elicited participants' views on the forensic medicine specialty and the potential introduction of a forensic medicine module into the medical curriculum, with variables similarly coded. The correct knowledge answers were awarded a score of 1, while incorrect and "do not know" responses were given a score of 0. The knowledge scores were calculated accordingly, with participants scoring over 75% classified as having "good" knowledge, those scoring below 50% classified as having "poor" knowledge, and those scoring between 51% and 74% classified as having "moderate" knowledge [11].

Data analysis

After data collection, data was entered in Excel spreadsheets (Microsoft® Corp., Redmond, WA, USA) for data cleaning, which involved identifying and removing duplicate entries, looking for outliers, and checking any missing data points. If less than 5% of the data for any variable was missing, we used pairwise deletion to ensure all available data was utilized in each analysis. For variables with more than 5% missing data, multiple imputation techniques were applied to estimate missing values based on observed patterns. In cases with significant missing data for a variable, the response was not involved in data analysis. A follow-up mechanism was put in place to encourage all participants to complete the study. Later, the data was coded and entered into Statistical Package for the Social Sciences (IBM SPSS Statistics for Windows, IBM Corp., Version 27.0, Armonk, NY) for analysis. All the categorical variables were presented in terms of count and frequencies, and the continuous variables were presented as mean and standard deviation. Inferential statistics like the Chi-square test were used to determine the association between categorical variables with a predetermined significance level set at p < 0.05. This comprehensive analytical approach aimed to extract meaningful insights and discern patterns within the dataset.

Ethical considerations

Ethical approval for this study was secured from Taif University's Committee reference no.: 45-091, with several ethical considerations meticulously addressed. Measures were implemented to uphold confidentiality and ensure informed consent. Prior to participation, participants were comprehensively briefed on the study's objectives, procedures, potential risks, and benefits. Their consent was solicited voluntarily without any coercion. Researchers pledged to safeguard participants' privacy by preserving their anonymity and sensitive data.

## Results

The study involved 92 Saudi medical students in regard to the evaluation of awareness and interest in forensic medicine as a career. Table 1 shows that the majority of the respondents are males (53.3%) while the rest are female (43.7%). The mean and standard deviation of the age of the respondents was 23.84±1.462.

**Table 1:** Demographic characteristics of the participants Gender is presented as count (n) and frequency (%). Age is presented as mean ± standard deviation (std).

Variable	Category	N (%)
Gender	Male	49 (53.3)
	Female	43 (46.7)
Age	mean±std	23.84±1.462

The results in Table 2 reveal an overwhelming majority (94.6%) that recognize forensic medicine as a part of the broader medical field. Furthermore, 87% had received formal education in forensic medicine, although 44.6% of these individuals believe that the education they received was inadequate. Respondents show good comprehension of some forensic medicine, for instance, 95.7% understand its role in assessing causes of death, and 91.3% recognize its importance in sexual assault cases. Most respondents know that forensic medicine plays a crucial role in assessing possible causes of death (95.7%). Notable gaps exist with nearly a quarter of respondents (22.8%) being uncertain whether forensic doctors are responsible for issuing death certificates, and 20.7% are unsure about their role as expert witnesses in court. The most prevalent understanding is that forensic medicine plays a crucial role in assessing possible causes of death (95.7%), indicating a strong foundational knowledge in this critical area.

**Table 2:** Assessment of knowledge regarding forensic medicine as a field and the responsibilities related to it. Data has been presented as count (n) and frequency (%).

Variable	Category	N(%)
Forensic medicine is a part of medicine:	No	4 (4.3)
	Yes	87 (94.6)
	I don’t know	1(1.1)
Have you received any formal education related to forensic medicine:	No	10(10.9)
	Yes	80(87)
	I don’t know	2(2.2)
Do you believe that the education you received regarding forensic medicine was adequate?	No	41(44.6)
	Yes	35(38)
	I don’t know	16(17.4)
Forensic medicine only deals with matters related to homicidal-suicidal cases?	No	66(71.7)
	Yes	20(21.7)
	I don’t know	6(6.5)
Forensic medicine helps in assessment of medical malpractice cases	No	5(5.4)
	Yes	66(71.7)
	I don’t know	21(22.8)
Forensic medicine has a role in assessment of possible causes of death	No	2(2.2)
	Yes	88(95.7)
	I don’t know	2(2.2)
Forensic medicine has a role in assessment of sexual assault-related crimes and its false fabrications	No	6(6.5)
	Yes	84(91.3)
	I don’t know	2(2.2)
Forensic medicine plays a pivotal role in matters dealing with disputed paternity, inheritance, and newborn swabs in maternal hospitals?	No	5(5.4)
	Yes	74(80.4)
	I don’t know	13(14.1)
Forensic doctor is responsible for issuing death certificates?	No	10(10.9)
	Yes	61(66.3)
	I don’t know	21(22.8)
Forensic doctor could be asked to attend court as an expert witness?	No	4(4.3)
	Yes	69(75)
	I don’t know	19(20.7)
Forensic doctor deals with cases related to physical/sexual abuse?	No	3(3.3)
	Yes	85(92.4)
	I don’t know	4(4.3)

Table 3 shows that more than three-quarters of the respondents (70.7%) believe that a forensic module would be beneficial, and 78.3% agree that it could clarify the roles played by a forensic physician. Furthermore, 77.2% of respondents feel that the module could assist in their future choice of medical residency. There is also strong consensus on the need for medical practitioners and students to understand medical liability laws (80.4%) and the concept of defensive medicine (79.3%). There was perceived educational importance with an overwhelming majority (88%) believing that a forensic medicine module could help students understand important medico-legal concepts. Despite nearly two-thirds of the students (63%) supporting participation in autopsies as part of the module, less than half (48.9%) expressed interest in pursuing forensic medicine as a career.

**Table 3:** Participants’ attitudes on forensic medicine specialty and introduction of a forensic medicine module into medical curriculum. Data has been presented as count (n) and frequency (%).

Variable	Category	N (%)
Do you believe that a forensic medicine module in your college would be beneficial?	No	27(29.3)
	Yes	65(70.7)
Forensic medicine module could help with clarifying the roles played by a forensic physician?	No	12(13)
	Yes	72(78.3)
	I don’t know	8(8.7)
Forensic medicine module could help student’s future choice of medical residency?	No	13(14.1)
	Yes	71(77.2)
	I don’t know	8(8.7)
Medical practitioners/medical students should be familiar with medical liability laws?	No	7(7.6)
	Yes	74(80.4)
	I don’t know	11(12)
Medical practitioners/medical students should be familiar with the concept of defensive medicine?	No	5(5.4)
	Yes	73(79.3)
	I don’t know	14(15.2)
Medico-legal implications of diagnostic-therapeutic choices may increase the risk of defensive medicine?	No	10(10.9)
	Yes	54(58.7)
	I don’t know	28(30.4)
Forensic medicine module could help students to understand important medico-legal concepts?	No	3(3.3)
	Yes	81(88)
	I don’t know	8(8.7)
In a forensic medicine module, students should be able to take part in autopsies?	No	13(14.1)
	Yes	58(63)
	I don’t know	21(22.8)
Autopsies could be helpful to medical students in learning human anatomy?	No	9(9.8)
	Yes	66(71.7)
	I don’t know	17(18.5)
Are you interested in choosing forensic medicine as a career?	No	45(48.9)
	Yes	47(51.1)

Table 4 shows that personal interest (39.1%) was the most significant factor in the choice of forensic medicine as a specialty, while financial rewards (19.6%) and job opportunities (19.6%) are less commonly seen as incentives. More than a third of the students, 35.9%, explicitly stated they were not interested in forensic medicine. In terms of influential factors aiding the decision to choose forensic medicine, lectures are the most impactful, with nearly three-quarters (73.9%) of the students acknowledging their importance. Other factors like conferences (46.7%) and social media (29.3%) are less influential, while television (19.6%) has the least impact.

**Table 4:** Factors influencing the choice of forensic medicine as a specialty among medical students. Data has been presented as count (n) and frequency (%).

Variable	Response	Category	N (%)
Which of the following factors do you believe could help you choose forensic medicine as a specialty?	Nature of the work	No	62 (67.4)
		Yes	30 (32.6)
	Personal interest	No	56 (60.9)
		Yes	36 (39.1)
	Financial rewards	No	74 (80.4)
		Yes	18 (19.6)
	Work hours	No	66 (71.7)
		Yes	26 (28.3)
	Job opportunities	No	74 (80.4)
		Yes	18 (19.6)
	Not interested in forensic	No	59 (64.1)
		Yes	33 (35.9)
Which of the following do you believe has influential factors in aiding medical students into choosing forensic medicine as a career?	Lecture	No	24 (26.1)
		Yes	68 (73.9)
	Social media	No	65 (70.7)
		Yes	27 (29.3)
	Conference	No	49 (53.3)
		Yes	43 (46.7)
	TV	No	74 (80.4)
		Yes	18 (19.6)

**Figure 1:**
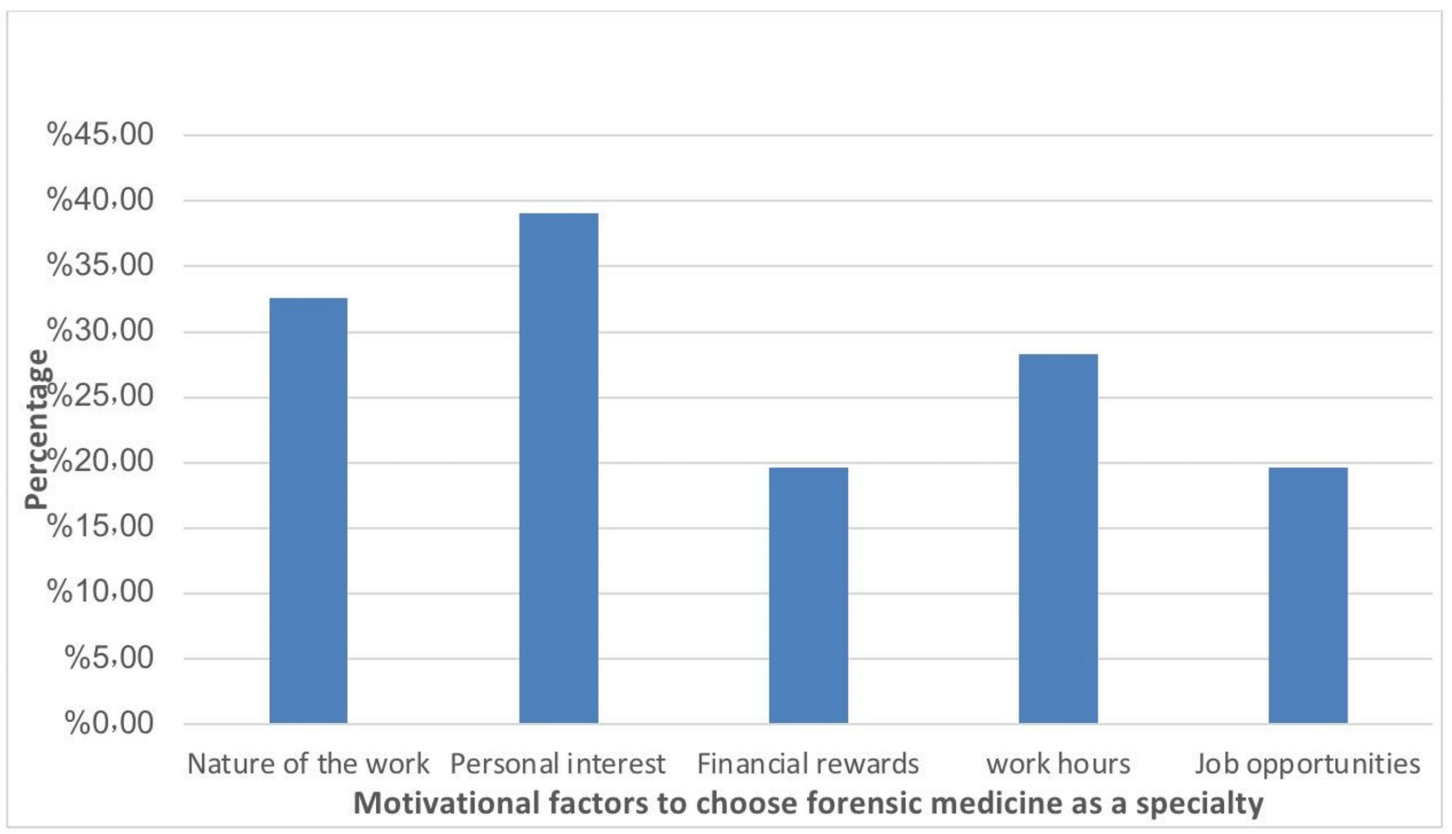
Motivator factors to choose forensic medicine

Figure 1 shows that personal interest stands out as the most significant motivator, with approximately 39% of students identifying it as a key factor. Following closely, the "nature of the work" motivates around 33% of students to pursue this specialty.

**Figure 2:**
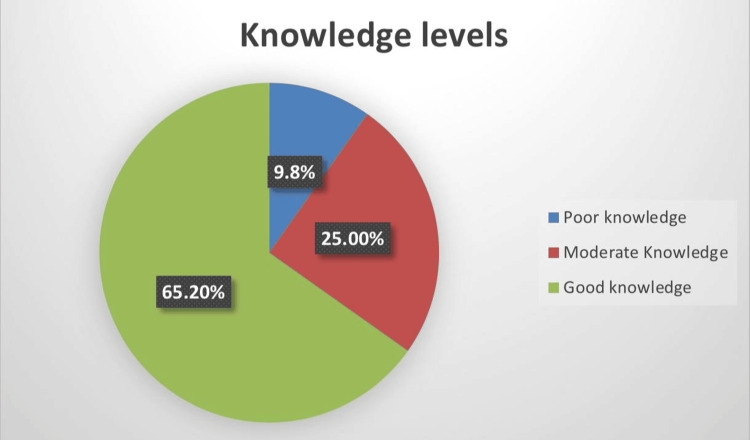
Knowledge level of the students toward forensic medicine

Figure 2 shows that the majority of the medical students, 60 (65.2%), demonstrated good knowledge, with scores exceeding 75%. Additionally, exactly a quarter of them, 23 (25.0%) exhibited fair knowledge, scoring between 50% and 75%. A smaller group, representing nine (9.8%) students showed poor knowledge with scores below 50%.

Table 5 shows no statistically significant differences between male and female respondents' views on various aspects of forensic medicine, as all p-values for the knowledge items were greater than 0.05. Overall, the analysis shows that gender does not significantly influence perceptions of forensic medicine in the variables studied.

**Table 5:** Relationship between gender and knowledge regarding forensic medicine as a field and the responsibilities related to it. Data has been presented as count (n) and frequency (%). The Chi-square test was used to determine statistical significance. The p-value is considered significant at p-value<0.05.

Variable	Category	Gender		P-value
		Male	Female	
Forensic medicine is a part of medicine:	No	3(75%)	1(25%)	0.386
	Yes	46(52.9%)	41(47.1%)	
	I don’t know	0(0.0%)	1(100%)	
Have you received any formal education related to forensic medicine?	No	7(70%)	3(30%)	0.181
	Yes	42(52.5%)	38(47.5%)	
	I don’t know	0(0.0%)	2(100%)	
Do you believe that the education you received regarding forensic medicine was adequate?	No	23(56.1%)	18(43.9%)	0.477
	Yes	16(45.70%)	19(54.3%)	
	I don’t know	10(62.5%)	6(37.5%)	
Forensic medicine only deals with matters related to homicidal-suicidal cases?	No	33(50%)	33(50%)	0.493
	Yes	13(65%)	7(35%)	
	I don’t know	3(50%)	3(50%)	
Forensic medicine helps in assessment of medical malpractice cases:	No	4(80%)	1(20%)	0.167
	Yes	37(56.1%)	29(43.9%)	
	I don’t know	8(38.1%)	13(61.9%)	
Forensic medicine has a role in assessment of possible causes of death:	No	1(50%)	1(50%)	0.309
	Yes	48(54.5%)	40(45.5%)	
	I don’t know	0(0.0%)	2(100%)	
Forensic medicine has a role in assessment of sexual assault-related crimes and its false fabrications:	No	4(66.7%)	2(33.3%)	0.257
	Yes	45(53.6%)	39(46.4%)	
	I don’t know	0(0.0%)	2(100%)	
Forensic medicine plays a pivotal role in matters dealing with disputed paternity, inheritance, and newborn swabs in maternal hospitals?	No	4(80%)	1(20%)	0.348
	Yes	37(50%)	37(50%)	
	I don’t know	8(61.5%)	5(38.5%)	
Forensic doctor is responsible for issuing death certificates?	No	8(80%)	2(20%)	0.089
	Yes	33(54.1%)	28(45.9%)	
	I don’t know	8(38.1%)	13(61.9%)	
Forensic doctor could be asked to attend court as an expert witness?	No	3(75%)	1(25%)	0.407
	Yes	38(55.1%)	31(44.9%)	
	I don’t know	8(42.1%)	11(57.9%)	
Forensic doctor deals with cases related to physical/sexual abuse?	No	3(100%)	0(0.0%)	0.256
	Yes	44(51.8%)	41(48.2%)	
	I don’t know	2(50%)	2(50%)	

## Discussion

Forensic medicine plays a critical role in the justice system, requiring a specialized understanding of legal and medical principles [12]. The integration of forensic medicine into medical education is essential to ensure that future healthcare professionals are adequately prepared to contribute to legal processes involving medical evidence [13]. Our study aimed to evaluate the medical students’ attitudes and knowledge regarding forensic medicine in Saudi Arabia. Our results showed that an overwhelming majority, 94.6%, agreed that forensic medicine is a fundamental part of the medical field. The high agreement among students suggests that future physicians see forensic knowledge as integral to their responsibilities, ensuring that they can handle legal aspects of patient care effectively. A similar study by Vignesh and Channabasappa among medical students found that the vast majority (92.3%) felt that forensic medicine, particularly autopsies, is essential for medical students [14]. This shows a universal recognition of the subject’s importance in medical education.

Further, our study revealed that 87% of the medical students have received formal education related to forensic medicine, though only 38% feel that the education was adequate, while 44.6% of students felt that the education they received regarding forensic medicine was inadequate. This highlighted a potential gap in the curriculum that could impact their preparedness to handle medico-legal cases. A similar study by Ansari et al. found even higher levels of dissatisfaction, with 57% of students expressing that their forensic medicine education was insufficient and in need of further enhancement [15]. The inadequate training in forensic medicine may leave future doctors underprepared to manage medico-legal cases.

The surveyed medical students demonstrated a strong acknowledgment of the importance of forensic medicine in medical education. Nearly three-quarters of the medical students (70.7%) believe it would be beneficial to incorporate the forensic medicine module in their curriculum. Furthermore, an overwhelming majority (88%) of the medical students agree that such a module would enhance their understanding of critical medico-legal concepts, underlining the perceived interest in knowledge. Our study further found that adequate exposure to forensic medicine may significantly influence students' professional paths by potentially increasing interest in the field, this is depicted by more than three-quarters of the medical students (77.2%) asserting that the forensic medicine module would shape their decision on medical residency. A similar study conducted among medical students at Dammam University found that the majority expressed a strong interest in forensic medicine and believed that its inclusion in their education had a positive impact on their overall medical training [7].

The surveyed medical students gave some compelling factors influencing their decision to pursue forensic medicine as a specialty. Among these, personal interest (39.1%) and the nature of the work (32.6%) emerged as the leading motivators. In contrast, financial rewards were less impactful, with only 19.6% of students citing them as a driving force behind their choice. Contrary to our findings, a study by Levaillant et al. revealed that lifestyle and work-life balance were the key motivating factors for specialty choice [16]. These disparities could be attributed to differences in cultural values and educational environments. This highlighted the significance of passion and the unique aspects of the field as key determinants. Further, our study revealed that lecturers played a pivotal role in shaping career decisions, with an impressive 73.9% of students recognizing their influence, highlighting the profound effect of academic guidance on career aspirations. Conversely, social media exerted a relatively minor influence, with just 29.3% of students considering it an important factor in their career choice. These findings agree with a different study that the decision to pursue forensic medicine is predominantly shaped by academic exposure and intrinsic motivations, rather than external influences [17].

Our study reveals a strong understanding of forensic medicine with nearly two-thirds (65.2%) of the medical students demonstrating good knowledge and exactly a quarter (25%) of the students having a moderate level of knowledge. The findings in our study reveal a higher level of knowledge among the students compared to a study conducted among Umm Al-Qura medical students, where only 17.8% demonstrated good knowledge, 23.2% had moderate knowledge, and the majority (59%) had poor knowledge [10]. The differences could be attributed to several factors, including variations in the curriculum and possibly the emphasis placed on forensic medicine in different medical schools.

The study revealed no statistically significant differences between gender and knowledge of forensic medicines (p-value>0.05) across all knowledge items. Suggesting that both male and female students possess similar levels of knowledge and misconceptions regarding forensic medicine, with no significant gender-based differences in understanding the field and its responsibilities. A study by Streb et al. that specifically aimed to determine gender differences in the choice of forensic medicine as a specialty found no statistically significant difference between gender and knowledge or specialty preference, thereby aligning with our findings [18].

Nevertheless, this study was marred by a few limitations one of them being the use of a descriptive approach, which allows for the identification of relationships between attributes but does not establish causality. The reliance on online surveys presents another limitation, as it introduces the potential for bias due to respondents possibly recording their answers accurately without verification. Moreover, the study's findings are specific to the Taif University students, limiting the generalizability of the conclusions to the broader population of Saudi Arabia.

## Conclusions

The majority of the medical students in this study demonstrated a high level of awareness of forensic medicine. Additionally, there was a strong consensus among medical students on the importance of forensic medicine and the necessity of incorporating it into the curriculum. However, despite the high level of recognition for forensic medicine, there remains a significant gap in the adequacy of current education, as many students feel underprepared for medico-legal responsibilities. Personal interest and the nature of the work are key motivators for students considering this specialty, with academic exposure, particularly through lectures, playing a pivotal role in shaping career decisions. The study also highlights the absence of significant gender differences in knowledge and attitudes toward forensic medicine. The study recommends that medical schools should incorporate a comprehensive forensic medicine module into the curriculum to address the significant gap in education.

## Appendices

**Table 6:** Personal information

Question	Options
Gender	- Female
	- Male
Age	(Open-ended)

**Table 7:** Knowledge regarding forensic medicine as a field and the responsibilities related to it: (11 questions)

Variable	Category	n(%)
Forensic medicine is a part of medicine	No	
	Yes	
	I don't know	
Have you received any formal education related to forensic medicine?	No	
	Yes	
	I don't know	
Do you believe that the education you received regarding forensic medicine was adequate?	No	
	Yes	
	I don't know	
forensic medicine only deals with matters related to homicidal-suicidal cases?	No	
	Yes	
	I don't know	
Forensic medicine helps in assessment of medical malpractice cases	No	
	Yes	
	I don't know	
Forensic medicine has a role in assessment of possible causes of death?	No	
	Yes	
	I don't know	
Forensic medicine has a role in assessment of sexual assault related crimes and its false fabrications?	No	
	Yes	
	I don't know	
Forensic medicine plays a pivotal role in matters dealing with disputed paternity, inheritance, and newborn swab in maternal hospitals?	No	
	Yes	
	I don't know	
Forensic doctor is responsible for issuing death certificates?	No	
	Yes	
	I don't know	
Forensic doctor could be asked to attend court as an expert witness?	No	
	Yes	
	I don't know	
Forensic doctor deals with cases related to physical/ sexual abuse?	No	
	Yes	
	I don't know	

**Table 8:** Participant opinion on forensic medicine specialty and introduction of a forensic medicine module into medical curriculum: (12 questions)

Variable	Category	n(%)
Do you believe that a forensic medicine module in your college would be beneficial?	No	
	Yes	
Forensic medicine module could help with clarifying the roles played by a forensic physician?	No	
	Yes	
	I don't know	
Forensic medicine module could help student’s future choice of medical residency?	No	
	Yes	
	I don't know	
Medical practitioners/ students should be familiar with medical liability laws?	No	
	Yes	
	I don't know	
Medical practitioners/ students should be familiar with the concept of defensive medicine?	No	
	Yes	
	I don't know	
Medico-legal implications of diagnostic-therapeutic choices may increase the risk of defensive medicine?	No	
	Yes	
	I don't know	
Forensic medicine module could help students to understand important medico-legal concepts?	No	
	Yes	
	I don't know	
In a forensic medicine module, students should be able to take part in autopsies?	No	
	Yes	
	I don't know	
Autopsies could be helpful to medical students in learning human anatomy?	No	
	Yes	
	I don't know	
Are you interested in choosing forensic medicine as a career?	No	
	Yes	
Which of the following factors do you believe could help you choose forensic medicine as a specialty: [ choose all that applies]		
Nature of the work. (dealing with dead, crimes, medical malpractice, etc.)		
Personal interest.		
Financial rewards.		
Work hours.		
Job opportunities.		
Not interested in forensic medicine.		
Which of the following do you believe has influential factors in aiding medical students into choosing forensic medicine as a career: [choose all that applies]:		
Lectures.		
Social media.		
Conferences and workshops.		
Tv and radio.		
Not interested in forensic medicine.		
